# Purification of influenza virus‐like particles using sulfated cellulose membrane adsorbers

**DOI:** 10.1002/jctb.5474

**Published:** 2017-12-16

**Authors:** Sofia B Carvalho, A Raquel Fortuna, Michael W Wolff, Cristina Peixoto, Paula M Alves, Udo Reichl, Manuel JT Carrondo

**Affiliations:** ^1^ iBET, Instituto de Biologia Experimental e Tecnológica Oeiras Portugal; ^2^ Instituto de Tecnologia Química e Biológica António Xavier Universidade Nova de Lisboa Oeiras Portugal; ^3^ Max Planck Institute for Dynamics of Complex Technical Systems Magdeburg Germany; ^4^ Institute of Bioprocess Engineering and Pharmaceutical Technology University of Applied Sciences Mittelhessen Gießen Germany; ^5^ Otto von Guericke University Magdeburg Magdeburg Germany; ^6^ Departamento de Química, Faculdade de Ciências e Tecnologia Universidade Nova de Lisboa Caparica Portugal

**Keywords:** downstream processing, membrane adsorption chromatography, sulfated cellulose, vaccine production, virus‐like particles

## Abstract

**BACKGROUND:**

Vaccines based on virus‐like particles (VLPs) are an alternative to inactivated viral vaccines that combine good safety profiles with strong immunogenicity. In order to be economically competitive, efficient manufacturing is required, in particular downstream processing, which often accounts for major production costs. This study describes the optimization and establishment of a chromatography capturing technique using sulfated cellulose membrane adsorbers (SCMA) for purification of influenza VLPs.

**RESULTS:**

Using a design of experiments approach, the critical factors for SCMA performance were described and optimized. For optimal conditions (membrane ligand density: 15.4 µmol cm^−2^, salt concentration of the loading buffer: 24 mmol L^‐1^ NaCl, and elution buffer: 920 mmol L^‐1^ NaCl, as well as the corresponding flow rates: 0.24 and 1.4 mL min^−1^), a yield of 80% in the product fraction was obtained. No loss of VLPs was detected in the flowthrough fraction. Removal of total protein and DNA impurities were higher than 89% and 80%, respectively.

**CONCLUSION:**

Use of SCMA represents a significant improvement compared with conventional ion exchanger membrane adsorbers. As the method proposed is easily scalable and reduces the number of steps required compared with conventional purification methods, SCMA could qualify as a generic platform for purification of VLP‐based influenza vaccines. © 2017 The Authors. *Journal of Chemical Technology & Biotechnology* published by John Wiley & Sons Ltd on behalf of Society of Chemical Industry.

## INTRODUCTION

Every season, influenza epidemic outbreaks raise serious health concerns and lead to substantial economic burdens.^1^ In fact, seasonal epidemics are responsible for 3 to 5 million cases of severe illness and up to 500 000 deaths annually worldwide.^2^ Moreover, the potential for the virus to cause pandemic outbreaks makes it a continuous public health threat that can result in millions of deaths.^1^ Vaccination remains the most effective and economical way to prevent and control infection for both seasonal and pandemic strains. However, due to antigenic drift and shift, seasonal vaccines need to be reviewed annually. Another concern relates to the poor efficacy of seasonal vaccines in the case of pandemics.^3^ The need to increase manufacturing capacity and flexibility, as well as decrease time to deliver vaccines, critical in the case of pandemics, has supported cell‐based vaccine production, in alternative to conventional egg‐based systems.^4^, ^5^

New vaccines produced using mammalian and insect cell lines that have been recently licensed^5^ and several platforms, including virus‐like particles (VLPs), are under development as candidates for both seasonal and pandemic Influenza virus.^3^, ^5^, ^6^, ^7^, ^8^, ^9^ In fact, VLPs hold great promise as vaccine candidates and have been long established for hepatitis B and human papillomavirus vaccines.^10^, ^11^ The potential of these platforms, together with the increasing demands on safety and quality control in vaccine manufacturing stresses the need for new downstream processing (DSP) methods for their purification.^12^ Influenza VLPs are being produced using different expression systems (mammalian, plant or insect cell cultures), which implies different purification strategies.^13^ Insect cell‐baculovirus expression system is a promising strategy to replace traditional egg and cell‐based systems. Advantages are related with short production times, high production yields and a straightforward scale‐up, maintaining efficiency.^14^ There are several reports describing different approaches to produce and purify influenza VLPs using insect cells. Most of the downstream strategies rely on traditional methods such as centrifugation, sucrose or iodixanol gradient ultracentrifugation.^13^, ^15^, ^16^ These methods are cumbersome and not easily scalable and most of the described purification strategies are not complete processes or do not cope with the purity specifications for human application. Novavax's influenza VLP vaccine manufacturing process combines these unit operations with ion exchange chromatography, ultrafiltration or diafiltration.^17^ In fact, there are several separation technologies available for DSP of biopharmaceuticals, which often include bead‐based chromatography.^18^, ^19^ However, this technique has several drawbacks, including high pressure drop across the packed bed, slow intraparticle diffusion, high process times and difficulties in scale‐up.^20^ Membrane‐based chromatography processes overcome some of the limitations associated with packed beds and have been increasingly applied for bioprocessing of large biomolecules, such as viruses and VLPs.^19^ Mass transfer constraints are improved through fully convective transport, which is possible due to the large pore size of membranes, and direct availability of the specific ligands on the membrane surface. These factors decrease process time and, together with low void volumes, reduce process and equipment costs. Moreover, membrane adsorbers can be used as disposable units, eliminating column packing, cleaning, regeneration and validation efforts.^19^, ^20^, ^21^ Several chromatographic membranes have been applied for the purification of biopharmaceuticals, including whole virus particles (WVPs) and VLPs.^19^ For influenza WVPs purification, the use of traditional cation and anion exchange membranes has been tested.^22^, ^23^ However, using only this approach, protein and DNA contamination levels exceeded acceptable limits for manufacturing of vaccines for human use. As an alternative, the use of pseudo‐affinity sulfated cellulose membrane adsorbers (SCMA) has been reported for DSP of influenza A WVPs. In particular, SCMA considerably improve DNA and total protein removal, show better strain robustness and productivity.^4^, ^23^, ^24^, ^25^

VLPs resemble native viruses by displaying the membrane proteins on their envelope.^26^ The similarity between both allows the transfer of processes established for DSP of WVPs to VLPs' purification. However, there are several factors that increase the complexity of VLPs' purification processes: lack of proper analytical tools,

$$\text{ionic capacity}=\frac{\begin{array}{c} \left[\left(V_{\text{load}}-V_{\text{void}}\right){\text{NaOH}}_{\text{conductivity}}-{\text{NaOH}}_{\text{peak area}}\right]{\text{NaOH}}_{\text{molarity}}/{\text{NaOH}}_{\text{conductivity}} \end{array}}{V_{\text{membrane}}}$$

high heterogeneity and low stability compared with native viruses, and presence of baculovirus particles as process impurity. Baculovirus' rod‐shape makes the discrimination between them and VLPs difficult to achieve. Moreover, VLPs and baculovirus have a similar envelope, as both bud from the cell. These increase the challenges faced in the downstream processing, as most of the baculovirus can be co‐eluting with the VLPs.^13^, ^27^, ^28^ Accordingly, process conditions have to be optimized. In this context, the use of a design of experiments (DoE) approach allows rational and fast screening and optimization of factors impacting yield and contamination levels. Similar approaches have been successfully applied to investigate the separation of other biopharmaceutical products, using different chromatographic matrices.^18^, ^29^, ^30^, ^31^, ^32^, ^33^ For instance, Fortuna et al.,^34^employed a DoE strategy to investigate the influence of both matrix and process‐related factors on the purification of influenza WVPs derived from mammalian cell cultures.

This work presents the establishment of a pseudo‐affinity chromatography method to successfully purify influenza VLPs using SCMA. The use of SCMA resulted in superior performance in terms of product recovery and impurity removal compared with other conventional membrane adsorbers. The approach reported is therefore a step towards establishment of a generic platform for VLP purification.

## MATERIALS AND METHODS

### Membrane production, characterization and assembly

Unmodified reinforced cellulose discs with a pore size of 3–5 µm (provided by Sartorius Stedim Biotech GmbH, Germany) were sulfated according to Wolff et al. 23, 25, 35 adapting the reaction time and temperature to achieve ligand densities of 7.9, 11.8 and 15.5 µmol $O‐{\mathrm{SO}}_{3}^{‐}$ cm^‐2^. Briefly, for 3 reactions chlorsulfonic acid (1.2 mL; Sigma Adrich, Germany) was added dropwise to pyridine (30 mL, Sigma Aldrich, Germany) on ice. Subsequently, the solution was heated to 65 °C until all precipitated compounds were completely dissolved. To obtain the different ligand densities, the respective reactions were cooled to 42 °C (7.9 µmol cm^‐2^), 45 °C (11.8 µmol cm^‐2^), 55 °C (15.5 µmol cm^‐2^), cellulose discs (diameter: 13 mm) were added to the reaction mixture and incubated for 16 h, 3 h, and 3 h, respectively. Next, the discs were washed with water and stored in 20% ethanol until further usage. For all chromatography runs and determination of the ligand density (LD) five discs (total membrane area and volume 5.65 cm^2^ and 0.14 mL) were mounted in a 13 mm diameter stainless steel Swinney Filter Holder (Pall Life Sciences, Germany).

Ligand density of the studied membrane adsorbers was determined via ionic capacity (IC) measurement. IC was calculated by exchanging hydrogen ion (H^+^), previously bound to the membrane, for sodium ion (Na^+^). Briefly, the membrane was conditioned with 1 mol L^‐1^ HCl for 35 column volumes (CV) and then washed with ultrapure water until conductivity was below 0.05 mS cm^‐1^ or for a maximum of 250 CV. H^+^ was then titrated with 10 mmol L^‐1^ NaOH until conductivity reached the maximum or for 300 CV. The IC determination was made using 100% of Na^+^ breakthrough, given in terms of conductivity, as the area below the chromatogram curve and considering the void volume of the membrane adsorber device (Equation (1)):

### Virus‐like particles production and clarification

Cell culture and VLP production were performed as described by Carvalho et al. 28 Briefly, High Five Cells were cultured in Insect‐EXPRESS™ medium (Lonza, Basel, Switzerland) and infected with recombinant baculovirus (kindly provided by Redbiotec AG) encoding for hemagglutinin subtype H1 from Influenza A/Brisbane/59/2007 and M1 protein from A/California/06/2009 virus strains. Infection was performed at a cell concentration (CCI) of 2 × 10^6^ cells per mL, with a multiplicity of infection (MOI) of 1 IP per cell. Baculovirus titers were determined by MTT cell viability assay.^36^, ^37^ One tablet per 50 mL of cell culture of EDTA‐free Protease Inhibitor Cocktail (05056489001, Roche Diagnostics, Germany) and 50 U mL^‐1^ of Benzonase® (101654, Merck Millipore, Germany) were added to the cell culture approximately 12 h before harvest. Cells were harvested at a viability of 50–60%, which corresponds to approximately 48 h post‐infection. Clarification was performed by sequential depth filtration using a D0HC filter (MD0HC23CL3, Merck Millipore, Germany) and an Opticap XL150 Capsule with 0.5/0.2 µm pore size (KHGES015FF3, Merck Millipore, Germany). Clarified material was aliquoted and stored at −80 °C.

### Virus production and primary processing

Influenza virus A/PuertoRico/8/34 (H1N1) whole particles were produced in suspension MDCK.SUS2 cells cultivated in chemically defined medium (Smif8, Gibco, by contact through K. Scharfenberg, FH Oldenburg/Ostfriesland/Wilhelmshaven, Germany)^38^ and pre‐processed as described by Fortuna et al. 34

### Desalting and sterile filtration

Sample preparation before the purification step was required for both VLPs and WVPs. Buffer exchange to phosphate buffered saline (PBS) with the required NaCl concentration for the loading buffer (more details in the chromatography section) was performed with a HiPrep 26/10 desalting column (17‐5087‐01, GE Healthcare LifeSciences, Uppsala, Sweden). A Minisart filter unit (0.2 µm) was used for sterile filtration (16534, Sartorius Stedim Biotech, Göttingen).

### Experimental design

A set of experiments was generated using a 3‐level optimization Rechtschaffner design supported by the software MODDE Pro 11 (Sartorius Stedim Data Analytics AB, Sweden). The factors investigated (Table 1) were: ligand density (LD, µmol cm^−2^), salt concentration for loading and elution (respectively, NaCl_load_ and NaCl_elution_, mmol L^‐1^) and flow rate in the load and elution steps, (respectively, Q_load_ and Q_elution_, mL min^−1^); and the responses considered: loss in the flowthrough and product yield in the elution fraction (both given as relative amount of the total of HA loaded). Overall, 24 chromatographic runs were carried out, including three replicates of the center point. The data was fitted using partial least squares regression (PLS) according to a general second‐order polynomial equation (Equation S‐1, see Supporting information).^39^, ^40^

**Table 1:** Factors investigated, and respective levels, for the optimization of influenza virus‐like particles (VLPs) using sulfated cellulose membrane adsorbers (SCMA)

Factors	Abbreviation	Level		
		Low	center	high
Ligand density (µmol cm^−2^)	LD	7.9	11.8	15.4
Salt concentration for load (mmol L^‐1^)	[NaCl]_load_	20	40	60
Salt concentration for elution (mmol L^‐1^)	[NaCl]_elution_	200	600	1000
Flow rate for load (mL min^−1^)	Q_load_	0.2	0.4	0.6
Flow rate for elution (mL min^−1^)	Q_elution_	0.5	1.0	1.5

Based on the results of the experimental design (Table S‐1, see Supporting information) and using MODDE's Optimizer, a combination of factors (set point) that minimizes HA loss during the loading step and maximizes the yield of the elution step was chosen.

### Chromatography experiments

All chromatography experiments were carried out on an ÄKTA Pure 25 system (UNICORN™ 6.3 software, GE Healthcare Bio‐Sciences AB, Uppsala, Sweden) and monitored inline using UV absorbance (at 280 nm wavelength) and dynamic light scattering (DLS, NICOMP™ 380 at 633 nm wavelength, Particle Sizing Systems, Santa Barbara, CA, USA). Briefly, the SCMA were equilibrated with loading buffer and then loaded. After a wash step with the loading buffer, the membranes were eluted in a single step with elution buffer, sanitized with 0.5 mol L^‐1^ sodium hydroxide (for 15 min) and re‐equilibrated. Each set of membranes was used for a maximum of five times. PBS was used as basis buffer system for all chromatographic runs. The concentration of NaCl in the loading and elution buffer was adjusted according to the design matrix (Table S‐1, see Supporting information) or to the predicted set point (Table 2). The total void volume of the system (1 mL considering system and membrane device) is considerably bigger than the total membrane volume, therefore, every chromatography step lasted as long as required to have a correct buffer exchange to the required buffer (minimum 4 mL). For the experiments in the design matrix, the membranes were loaded with 5 mL of the desalted and filtered VLP material. Dynamic binding capacity at 20% breakthrough (DBC_20%_) was determined with the conditions of NaCl_load_, NaCl_elution_, Q_load_ and Q_elution_ predicted for the set point. Sample loading (10 mL) was monitored by absorbance and light scattering signal. In addition, the amount of hemagglutinin protein in the flowthrough fractions was quantified offline as described in the hemagglutination assay section. The DBC_20%_ and the chosen set point were experimentally verified with technical replicates (n = 3). For the set point, a loading challenge of 70% of the DBC_20%_ was used. Chromatographic purification performance with SCMA was compared with Sartobind® pico S and Sartobind® pico Q (both 0.08 mL bed‐volume) using the operating parameters described for the set point and technical replicates (n = 3). The flow rates were scaled down, keeping the residence time for the loading and elution step constant. The elution of the Sartobind® Q included three steps with elution buffers containing 600 mmol L^‐1^, 920 mmol L^‐1^ and 2 mol L^‐1^ NaCl. For Sartobind® S only the two last elution steps were performed.

Finally, as positive control, the SCMA performance with influenza WVPs was evaluated (n = 3) under the same set point operation conditions, loading 4 mL.

### Total protein quantification

To quantify the total protein present in each sample, the BCA Protein Assay Kit (23225, Thermo Fisher Scientific, USA) was used, following manufacturer's instructions. Bovine serum albumin (BSA) was used for the calibration curve (23209, Thermo Fisher Scientific, USA). Samples were diluted between 2‐ and 256‐fold to avoid interference with the method. A clear flat bottom 96‐well microplate (655101, Greiner Bio‐One GmbH, Germany) was utilized and the absorbance at 562 nm was measured on Infinite® M200 PRO NanoQuant (Tecan, Switzerland) microplate multimode reader.

### Total dsDNA quantification

Total dsDNA was determined according to the fluorescence method described by Opitz et al. 41 and using the Quant‐iT™ Picogreen® dsDNA reagent (P7581, Molecular Probes, USA). The standard curve was prepared with λ‐DNA (#D1501, Promega GmbH, Germany). The assay was carried out in a black 96‐well microplate, flat transparent (3915, Corning, USA) and the fluorescence (λ_excitation_ = 485 nm, λ_emission_ = 535 nm) was measured on the Infinite® M200 PRO NanoQuant (Tecan, Switzerland) microplate multimode reader.

### Hemagglutination assay

Hemagglutinin (HA) protein content was evaluated using two hemagglutination assays: a quantitative assay for VLPs and an activity assay for virus, respectively. Quantitative hemagglutination assay, for HA quantification of VLPs, was carried out based on the protocol described elsewhere^27^ with some modifications. Briefly, 50 µL or 66.7 µL, depending on the dilution factor, of PBS were added in each well of a clear, V bottom 96‐well microtiter plate (611 V96, Sterilin, USA). For each sample, two initial dilutions were performed, 1:2 and 1:3. 50 µL or 33.3 µL were added to the first well of each line and then two‐fold serial dilutions (50 µL of sample in an equal volume of PBS) were performed. The final 50 µL from the last dilution were discarded. Finally, 50 µL of 1% chicken erythrocytes (LOHMANN TIERZUCHT GmbH, Germany) were added to each well. The plate was incubated at 4 °C for at least 30 min without disturbance. As positive control, an internal standard purified and concentrated was used. The standard HA concentration was previously evaluated by SRID assay. The level of hemagglutination was inspected visually for all the wells and the highest dilution capable of agglutinating chicken erythrocytes was determined.

Influenza WVPs were quantified according to the method described by Kalbfuss et al. 42 Briefly, each sample was prepared in two different pre‐dilutions by adding PBS to 100 µL or 70.7 µL of virus sample to achieve a final volume of 200 µL. These were then diluted in series (1:2) with PBS in a U‐bottom 96‐well plate (650160, Greiner Bio‐One, Germany). In addition, an internal standard was included and measured at least twice. 100 µL of purified chicken erythrocytes (2 × 10^7^ cell mL^‐1^) were added to each well and the plates were allowed to incubate for at least 2 h, before evaluation.

### Transmission electron microscopy

TEM analysis was performed to analyze the presence, integrity and morphology of the VLPs before and after the SCMA purification step. Sample preparation was performed as follows: a drop (5 µL) of each sample was adsorbed onto a formvar coated 150 mesh copper grid from Veco (Science Services, Germany) for 2 min. Then, the grid was washed five times with sterile filtered dH_2_O, soaked in 2% uranyl acetate for 2 min and dried in air at room temperature (22 °C). A Hitachi H‐7650 120 Kv electron microscope (Hitachi High‐Technologies Corporation, Japan) was used to analyze the samples.

## RESULTS AND DISCUSSION

### Experimental design and optimization

The chromatographic purification of influenza VLPs reported in this work was optimized using a design of experiments (DoE) approach considering factors already identified as critical for the purification of influenza whole virus particles (WVPs) using sulfated cellulose membrane adsorbers (SCMA).^4^, ^23^, ^25^, ^34^ Taking into account the similarities between WVPs and VLPs, it is expected that these factors will be important. Table 1 summarizes the factors investigated: ligand density (LD), load and elution conductivity were already reported; load flow rate was assessed as it was observed that residence time is important for binding and further elution; elution flow rate has also an important role in product recovery.^43^, ^44^ The results showed that product recovery increases with increase of elution flow rate. This can be explained by the improvement of mechanical removal of VLPs from the membrane. Higher flow rates force the particles to detach from their binding sites and entrapment of these large particles into membrane pores is reduced. Although product concentration is usually taken into consideration in the experimental design, in this case it was left out of the investigation. Our strategy intends to use VLPs' clarified bulks (always with low HA concentrations) as loading product for the SCMA. From previous knowledge, HA concentration is similar (the same order of magnitude) among different influenza VLPs, and using the insect cells/baculovirus expression system. However, this factor should be evaluated in the future if one intends to use a SCMA train at a different stage of the downstream process.

**Figure 1:**
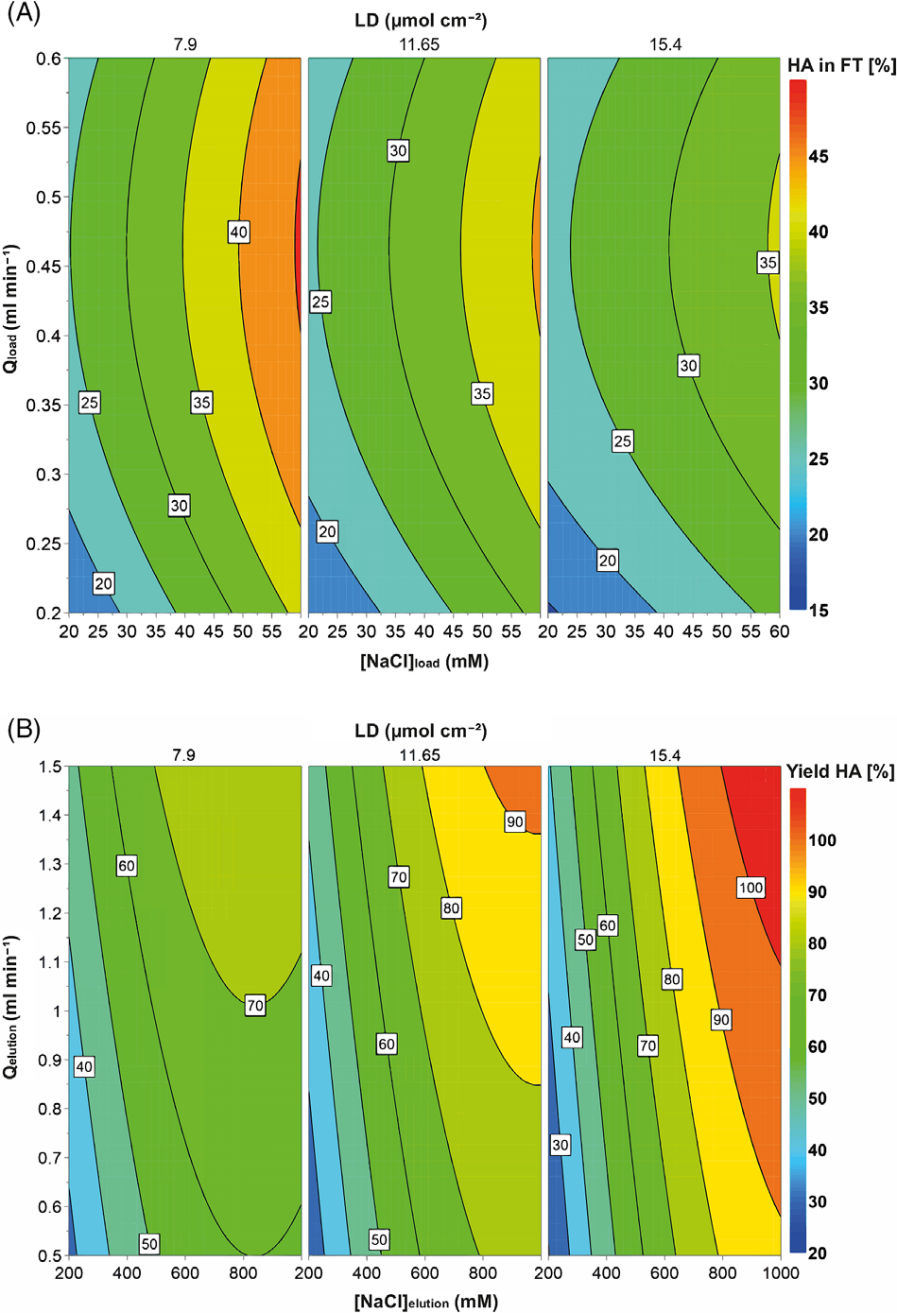
Contour plots generated with MODDE Pro 11, according to the model predicted (Equations S‐2 and S‐3, in Supporting information) for HA loss in the flowthrough (A) and yield in terms of HA (B). Membrane ligand density (LD), salt concentration for loading and elution (respectively NaCl_load_ and NaCl_elutio_n) and flow rate in the load and elution steps (respectively Q_load_ and Q_elution_).

The initial design considered several responses: HA recovery yield (in the product fraction), loss of HA (in the flowthrough fraction) and contaminant removal (DNA, total protein and baculoviruses) from the product fraction. The goal was to establish a set point with the highest HA recovery and the lowest value for all the other responses. However, as a bind‐elute strategy was used, total protein and DNA presented low values in the product fraction, often below the limit of detection (LOD) of the quantification methods. Moreover, to minimize baculovirus content, the set point would be pushed towards higher HA losses in the flowthrough and lower recovery yields. The best operation set point was then adjusted to minimize the HA loss in the flowthrough and maximize the product yield, excluding the impurities content from the optimization objectives. The contour plots on Fig. 1(A)–(B), graphically represent the mathematical fitting of the HA loss and yield to the factors that describe them (Equations S‐2 and S‐3, in Supporting information). The regression model was significant for both responses according to the analysis of variance (Table S‐II, in Supporting information).

In the case of HA loss in the flowthrough of the loading step (Fig. 1(A)), the factors related to elution ([NaCl]_elution_ and Q_elution_) were excluded since these are only relevant in the subsequent step. Within the range of the investigated factors, the model predicts HA loss between 15% and 45%. Lower Q_load_, resulting in higher residences times, allows more time for the interaction between the VLPs and the chromatographic matrix to be established; while higher ligand densities increase the availability of ligands for the particles to interact with, as well as the strength of the interaction. The same trends for LD were observed by Fortuna *et al.,* 34 with influenza WVP, although they investigated a higher range (14–25 µmol cm^−2^). Similar to what was observed in this work, for optimal LD the salt tolerance during load is the highest and therefore the NaCl_load_ can be higher without compromising the reduced particle losses.

Regarding the HA yield, all five factors (including linear combinations of these) describe this response. The contour plot (Fig. 1(B)) predicts, for a fixed Q_load_ = 0.2 mL min^−1^, yield values from 30% up to full recovery of the HA loaded. It is possible to confirm that, as expected from the mass balances, conditions that reduce loss of HA in the flowthrough also benefit product yield in the elution step, i.e. higher ligand densities and lower [NaCl]_elution_., in addition to a low Q_load_. Concerning the factors directly related to the elution step, the higher range of both [NaCl]_elution_ and Q_elution_ results in better yield values. These conditions lead to a fast elution and concentrated fraction.

Table 2 summarizes the predicted set point and the conditions implemented to test the membrane performance experimentally. The only restriction to the suggested set point was in the LD, as only membranes with a certain LD were available, the closest value was selected. The experiments were carried out with membrane adsorbers with the highest LD (15.4 µmol cm^−2^), which is still within the standard deviation of the predicted value.

**Table 2:** Set point predicted by Monte Carlo simulation (resolution 16, 10 000 simulations per point, 95% confidence level) and experimentally implemented. The predicted values are presented as average ± standard deviation

**Factors**	Predicted	Experimental
LD (µmol cm^−2^)	14.7 ± 1.2	15.4
[NaCl]_load_ (mmol L^‐1^)	24 ± 16	24
[NaCl]_elution_ (mmol L^‐1^)	920 ± 307	920
Q_load_ (mL min^−1^)	0.24 ± 0.15	0.24
Q_elution_ (mL min^−1^)	1.4 ± 0.2	1.4

### Validation of the optimal set point

Using the implemented set point described in Table 2, SCMA dynamic binding capacity at 20% breakthrough (DBC_20%_) for the influenza VLPs was calculated. In these conditions, the DBC_20%_ was 78 ± 17 ng_HA_ ml^−1^ (n = 3) with variations corresponding to the error associated with the hemagglutination assay. This value is lower than the one obtained for influenza virus (data not shown) which is expected, taking into account the differences in the strains, heterogeneity of VLPs, when compared with native virus, and also the presence of baculovirus, which compete for the binding sites. Moreover, the expression system was not the same, which affects the DNA and total protein levels and, consequently, impacts DBC.

**Figure 2:**
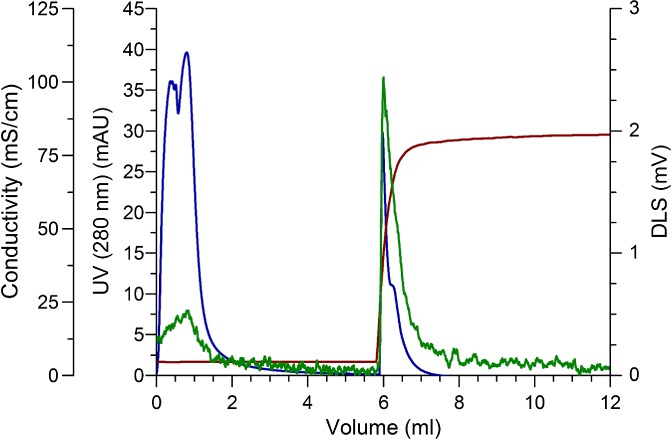
Representative chromatogram (n = 3) of the purification of influenza VLPs using SCMA. Red line denotes conductivity, blue line UV signal measured at 280 nm, and the green line DLS signal.

**Figure 3:**
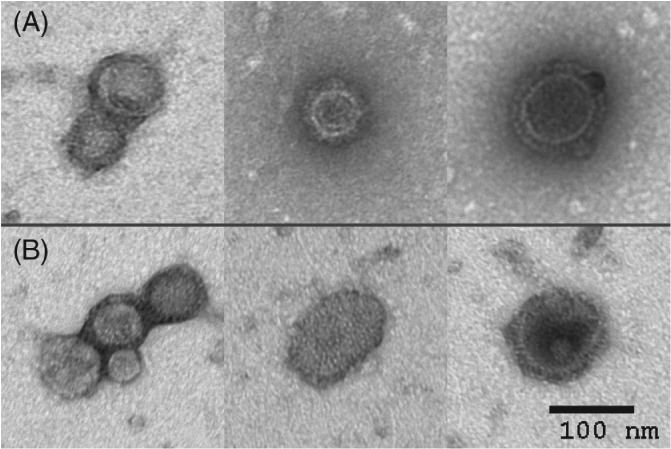
Transmission electron microscopy micrograph of influenza VLPs from the initial bulk sample (A) and from the elution step of the SCMA purification process (B). Scale bar indicates 100 nm.

To evaluate experimental set point performance, SCMA was challenged with a load equivalent to 70% of the DBC_20%_ (0.31 µg_HA_), the HA yield was 79.7 ± 5.8% and no losses of HA were detected in the flowthrough fraction. Since the loaded volume was not the same as the one used for the DoE experiments, it is not possible to directly compare the responses (HA yield and losses in the) with the predicted ones. However, any batch chromatography purification process, in bind and elute mode, will be carried out using a load corresponding to a reasonable challenge of the DBC, which validates the load conditions mentioned above. The representative chromatograms corresponding to these three technical replicates are represented in Fig. 2. The UV, measured at 280 nm, and the DLS signals distinguish where VLPs and baculovirus are co‐eluting. In the flowthrough fraction UV signal is high (of the same magnitude as the elution peak), while DLS remains low. In fact, offline assays reveal that no HA is present in the flowthrough, so the UV signal corresponds to process impurities and not to influenza VLPs. UV and DLS signals are concomitant in the elution, starting to increase with the conductivity. The presence of VLPs in the elution step was confirmed not only by the higher DLS signal but also by the HA quantification. VLPs' purity was assessed by the total protein/HA and total DNA/HA ratios, before and after SCMA purification. The values for total protein were 0.39 ± 0.04 mg_tot.prot._ µg_HA_^‐1^ and $0.07\pm 0.03\;{\text{mg}}_{\text{tot.prot.}}\;µ{\text{g}}_{{\text{HA}}^{-1}}$, before and after purification, respectively. For DNA they were $0.11\pm 0.04\;µ{\text{g}}_{\text{DNA}}\;µ{\text{g}}_{{\text{HA}}^{-1}}$ and <0.02 µg_DNA_ µg_HA_^‐1^, before and after purification, respectively. These values correspond to a total protein removal of 82.2% and a total DNA removal above 78.9% suggesting a high purity level. Moreover, to evaluate the presence, integrity and morphology of the VLPs TEM analysis was performed (Fig. 3). Initial bulk (Fig. 3(A)) and elution steps of the SCMA purification process (Fig. 3(B)) samples were analyzed. VLPs' morphology is maintained after the purification process; their size, although heterogeneous, and spherical shape are similar for both samples evaluated. In addition, it is possible to observe that particles contain the ultrastructural details characteristic of influenza HA spikes.^45^, ^46^ These results revealed that SCMA chromatography as a single unit operation does not have an impact on VLPs.

### SCMA process performance

The potential of SCMA as chromatographic matrix for a downstream processing train for influenza VLPs was compared with the membrane adsorbers Sartobind® Q and Sartobind® S. HA recovery yield, losses in the flowthrough, DNA and total protein impurity levels were analyzed (Table 3). To compare these membranes, it is important to take into account several parameters: pore size, chemical properties and configuration, as well as ligand density. All membranes have similar pore sizes of >3 µm, but they present different ligand type and density. Both Sartobind® membranes have a cellulose‐based macro‐porous structure, which is then functionalized either with quaternary ammonium ligands (Q), being a strong anion exchanger; or with sulfonic acid ligands (S), being a strong cation exchanger. SCMA have the same backbone structure and a cation exchanger character resulting from the sulfate ligands, which are directly bound to the sugar ring of the cellulose, through a covalent bond. The ligand density also distinguishes them, with SCMA presenting higher ligand densities. Since Sartobind Q and Sartobind S matrices are both ion exchangers (IEX), the mechanism of loading and elution is regulated by ionic strength. As DoE and previous reports revealed,^23^, ^34^ SCMA purification of influenza particles (virus and VLPs) requires loading with low salt containing feed streams. Also, the same conditions can promote binding to the IEX membranes. Those conditions were evaluated, avoiding unnecessary optimization efforts and material consumption. Gradient runs were performed to optimize ionic strength for elution of the IEX membranes (data not shown). Using this information, buffer composition was determined for the elution steps performed. Interestingly, these were the same for S and Q membranes. The additional step performed for Q membrane with a higher ionic strength did not further improve virus recovery. The theoretical isoelectric point for the evaluated strain is 6.74. This value was calculated using the ExPASy ProtParam tool that assumes that all titrable aminoacid residues have the same pKa value in solution and in the protein environment. Due to product and process complexity it is very difficult to have a correct experimental value, as the overall charge of the particles can also change with the purification step. Nevertheless, considering the theoretical value, at the working pH (7.4) the VLPs have a negative charge. Therefore, as expected for the anion exchanger (Sartobind®Q), no losses in the flowthrough were observed. HA recovery was 47.4% which is a low yield, and significantly lower (Student's two‐tailed t‐test for paired samples, *P* = 0.016) than the one obtained with SCMA (79.7%). This might indicate VLPs entrapment in the membrane, even at high conductivities. Total protein recovered in the elution fraction was more than 2.4 times higher than the level obtained with SCMA (*P* = 0.008). DNA value is not compared as it was below LOD. In the case of Sartobind® S, HA losses in the flowthrough were significantly greater than zero (one sample Student's one tailed t‐test, *P* = 0.017), around 23.8%, which can be explained by the electrostatic repulsion between the negative charges of the virus and membrane ligands. Therefore, the recovery in the elution fraction was 45.9 %, significantly lower than SCMA (Student's two‐tailed t‐test for paired samples, *P* = 0.012). DNA level was also below LOD and the ratio total protein/HA was more than 1.7 times higher (*P* = 0.004). It can be discussed if changing pH will improve Sartobind® S performance but these VLPs are less stable at low pH than at neutral pH. Moreover, SCMA can be used as a platform for influenza VLPs purification, applicable for several strains, which invalidates pH‐dependent processes. Finally, direct comparison of Sartobind® S and SCMA also shows that the interaction between the virus and the sulfate ligand in the latter case, is not solely of an electrostatic nature and rather relies on a pseudo‐affinity interaction with the hemagglutinin molecules in the VLPs surface. Although both membranes have negatively charged groups, sulfated cellulose resembles Heparin, a naturally occurring glycosaminoglycan, to which several hemagglutinins are known to have affinity.^47^ The chromatography strategy presented here takes advantage of this affinity to selectively retain VLPs presenting hemagglutinin in their surface and therefore purifying them.

**Table 3:** Comparison between VLPs purification with Sartobind® Q and S and SCMA and between VLPs and virus purification using SCMA

Product	Matrix	HA in flowthrough (%)	Yield HA (%)	DNA (µg_DNA_ µg_HA_^−1^)	Total protein (mg_tot.prot._ µg_HA_^−1^)
VLPs	Sartobind® Q	0.0 ± 0.0 (<LOD)	47.4 ± 0.0	<LOD	0.26 ± 0.03
	Sartobind® S	23.8 ± 7.8	45.9 ± 4.0	<LOD	0.18 ± 0.05
	SCMA	**0.0 ± 0.0 (<LOD)**	**79.7 ± 5.8**	**<LOD**	**0.07 ± 0.03**
Whole virus	SCMA	2.4 ± 0.1	64.0 ± 0.1	0.0038 ± 0.0003	0.013 ± 0.001

SCMA performance was also evaluated for influenza WVP as a positive control (Table 3). The recovery yield was 64% and there was around 2.4% of HA in the flowthrough. The results were close to the ones obtained with VLPs. Differences in yield and HA losses in the flowthrough can be explained by different affinity for the ligand. DNA levels were measurable for virus fraction but still low (0.0038 µg_DNA_ µg_HA_^−1^). In the case of total protein, the value obtained for virus was 5.38 times lower than the one obtained for VLPs. The small differences can derive from the different production systems (insect vs mammalian cells), virus strain and previous downstream treatment.

## CONCLUSIONS

Herein, a downstream processing strategy to purify influenza VLPs using sulfated cellulose membrane adsorbers (SCMA) is reported for the first time.

The DoE results showed that, for an optimized process, high ligand densities, low flow rate and salt concentration for the load and high flow rate and salt concentration for elution should be used. The evaluated responses were established to define a set point with the highest HA recovery and the lowest value of HA in the flowthrough. Using these conditions, DBC_20%_ (78 ± 17 ng_HA_ mL^−1^) was determined and the performance of SCMA using the predicted set point was evaluated with a 70% challenge of DBC_20%_. The recovery obtained was 79.7% ± 5.8% and no losses of HA in the flowthrough were observed. Importantly, this system does not have an impact on the morphology of the VLPs as confirmed by TEM analysis.

SCMA were compared with conventional membrane ion exchangers, Sartobind® S and Sartobind® Q, and were superior not only in terms of recovery yields and losses in the flowthrough but also concerning impurity removal. As positive control, it was additionally confirmed that these specific SCMA under the optimal conditions for VLPs, are also suitable for whole influenza virus particles purification.

This approach represents a step towards improvement and efficient development of purification techniques for VLPs. Moreover, this DSP unit operation is easily scalable and allows a reduced number of purification steps, overall supporting the use of SCMA as platform for purification of influenza particles.

## AUTHOR CONTRIBUTIONS

S.C., A.R.F., M.W. and C.P. conceived the study. S. C. and A.R.F. designed and performed VLPs and virus production and the downstream processing studies. M.W. produced the membrane adsorbers. C.P., M.W. and M.C. directed the project. S.C. and A.R.F. wrote the manuscript with contributions from all authors.

## Supporting information

SUPPORTING INFORMATION
