# Supplementary material for: Purification of influenza virus‐like particles using sulfated cellulose membrane adsorbers
Source: J Chem Technol Biotechnol. 2017 Dec 16;93(7):1988–96. doi: 10.1002/jctb.5474 (PMC6033026; doi:10.1002/jctb.5474)
Supplement: Supplementary file 1 — SUPPORTING INFORMATION [file JCTB-93-1988-s001.docx]

**SUPPORTING INFORMATION**

**Purification of influenza virus-like particles using sulfated cellulose membrane adsorbers**

**Sofia B. Carvalho, A. Raquel Fortuna, Michael Wolff, Cristina Peixoto, Paula M. Alves, Udo Reichl, Manuel J.T. Carrondo**

To whom correspondence should be addressed:

E-mail (C.P.): [peixoto@ibet.pt](mailto:peixoto@ibet.pt)

E-mail (M.W.): mwolff@mpi-magdeburg.mpg.de

**Table of contents**

Experimental Section 2

Design matrix 3

Model fitting and statistical analysis 4

**Experimental Section**

**Baculovirus quantification**

To quantify baculovirus viral copies in each sample, viral DNA was extracted and purified using the High Pure Viral Nucleic Acid Kit (11858874001, Roche Diagnostics, Germany) following manufacturer’s instructions. The number of genome containing particles were monitored by real time quantitative PCR (q-PCR) following the protocol described elsewhere [^1^](#_ENREF_1) with some modifications. Briefly, DNA samples were diluted 1:100 in water PCR grade (03315932001, Roche Diagnostics) and diluted again 1:4 with master mix. Master mix is prepared by diluting 1:2 the Light Cycler® 480 SYBR Green I Master (04707516001, Roche Diagnostics, Germany) and 0,5 µM of each primer. q-PCR reaction took place in a 96-well white plate (04729692001, Roche Diagnostics) using a LightCycler® 480 Instrument II (Roche Molecular Systems, Inc.).

**Nanoparticle tracking analysis**

After VLPs production and clarification, particle presence, concentration and size distribution were measured using the NanoSight NS500 (Nanosight Ltd, UK). Samples were diluted in D-PBS (14190-169, Gibco®, UK) so that VLPs concentration would be in the 10^8^–10^9^ particles/ml – the instrument’s linear range. All measurements were performed at room temperature (22 °C). Sample videos were analysed with the Nanoparticle Tracking Analysis (NTA) 2.3 Analytical software - release version build 0025. Capture settings (shutter and gain) were adjusted manually. For each sample 60-seconds videos were acquired and particles between 70 and 150 nm were considered.

**Design matrix**

Using MODDE 11, an experimental matrix was generated for a 3-level optimization Rechtschaffner design. **Table S-I** resumes the values for each investigated factor and the resulting responses for each experiment.

**Table S-I** - Design matrix (3-level Rechtschaffner design) implemented for the optimization of the chromatographic purification of influenza VLPs using sulfated cellulose membrane adsorbers (SCMA). Five responses (ligand density (LD), salt concentration for loading and elution (NaCl_load_ and NaCl_elution_, respectively) and flow rate in the load and elution steps elution (Q_load_ and Q_elution_, respectively)) and two responses (HA loss and HA yield) were investigated. The center points of the design are marked with a star (*) and outlier values, not considered for data fitting, appear between brackets.

| Exp. no. | LD | [NaCl]_load_ | [NaCl]_elution_ | Q_load_ | Q_elution_ | HA loss | HA yield |
| --- | --- | --- | --- | --- | --- | --- | --- |
|  | µmol cm^−2^ | mM | mM | ml min^−1^ | ml min^−1^ | % | % |
| 1 | 7.9 | 20 | 200 | 0.2 | 0.5 | 17 | 37.5 |
| 2 | 7.9 | 60 | 1000 | 0.6 | 1.5 | 41.7 | 97.3 |
| 3 | 15.4 | 20 | 1000 | 0.6 | 1.5 | 21.8 | (155.9) |
| 4 | 15.4 | 60 | 200 | 0.6 | 1.5 | 32.9 | 37.3 |
| 5 | 15.4 | 60 | 1000 | 0.2 | 1.5 | 30.9 | 112.9 |
| 6 | 15.4 | 60 | 1000 | 0.6 | 0.5 | 30.9 | 90.5 |
| 7 | 15.4 | 60 | 200 | 0.2 | 0.5 | (0) | 12.3 |
| 8 | 15.4 | 20 | 1000 | 0.2 | 0.5 | 10.9 | 103.9 |
| 9 | 15.4 | 20 | 200 | 0.6 | 0.5 | 23.2 | 19.9 |
| 10 | 15.4 | 20 | 200 | 0.2 | 1.5 | 16.7 | 53.4 |
| 11 | 7.9 | 60 | 1000 | 0.2 | 0.5 | 26.8 | 66.5 |
| 12 | 7.9 | 60 | 200 | 0.6 | 0.5 | 48 | 21.8 |
| 13 | 7.9 | 60 | 200 | 0.2 | 1.5 | 42.9 | 42.2 |
| 14 | 7.9 | 20 | 1000 | 0.6 | 0.5 | 21.4 | 21.8 |
| 15 | 7.9 | 20 | 1000 | 0.2 | 1.5 | 13.2 | 69.3 |
| 16 | 7.9 | 20 | 200 | 0.6 | 1.5 | 24.2 | 41.6 |
| 17 | 15.4 | 40 | 600 | 0.4 | 1 | 27.3 | 58.1 |
| 18 | 11.8 | 60 | 600 | 0.4 | 1 | 37.7 | 74 |
| 19 | 11.8 | 40 | 1000 | 0.4 | 1 | 36 | 102.6 |
| 20 | 11.8 | 40 | 600 | 0.6 | 1 | 25.9 | 56.7 |
| 21 | 11.8 | 40 | 600 | 0.4 | 1.5 | 28 | 80 |
| 22 | 11.8 | 40 | 600 | 0.4 | 1 | 32.6 | 78.3 |
| 23 | 11.8 | 40 | 600 | 0.4 | 1 | 34.9 | 92.3 |
| 24 | 11.8 | 40 | 600 | 0.4 | 1 | 31.4 | 69.6 |

**Model fitting and statistical analysis**

Each response Y is described as a function of each factor X and all possible factors interactions according to the general second order polynomial equation ((S-1)):

$Y=C_{0}+\sum_{i=1}^{n} C_{i}X_{i}+\sum_{i=1}^{n} C_{ii}X_{i}^{2}+\sum_{i,j=1}^{n} C_{i,j}X_{i}X_{j}$ (S-1)

where $C_{i}$ are the calculated regression coefficients, $n$ the total number of factors, and $j$ all other factors except $i$. All coefficients were scaled, centered and normalized to the variance of each response.[^2^](#_ENREF_2)

Equations S-2 and S-3 describe respectively HA loss and HA yield, excluding all terms found not-significant.

$HA loss=32.1-2.3\cdot LD+6.9\cdot\left[ NaCl \right]_{load}+2.1\cdot Q_{load}-3.9\cdot{Q_{load}}^{2}-1.6\cdot LD\cdot\left[ NaCl \right]_{load}$ (S-2)

$HA yield=71.3-4.8\cdot LD+2.8\cdot\left[ NaCl \right]_{load}+22.0\cdot\left[ NaCl \right]_{elution}-4.5\cdot Q_{load}+8.3\cdot Q_{elution}-9.3\cdot{\left[ NaCl \right]_{elution}}^{2}+7.0\cdot LD\cdot\left[ NaCl \right]_{elution}+6.3\cdot\left[ NaCl \right]_{load}\cdot\left[ NaCl \right]_{elution}+5.0\cdot\left[ NaCl \right]_{load}\cdot Q_{load}$ (S-3)

Analysis of variance (ANOVA) was used for the evaluation of the significance of the regression model of both responses (**Table S- II**). For both responses, the model is significant as attests the p-value (< 0.05), the F-value below the respective F_crit_ (F_crit_ ≤ 2.8) and the lack of fit (> 0.05). Furthermore, the explained variation (R^2^), predicted variation (Q^2^), model validity and reproducibility are within accepted ranges. [^2^](#_ENREF_2)

**Table S- II.** ANOVA for the proposed experimental design together with the predicted variation (Q^2^), the validity, and the reproducibility of the model for both responses.

| Response | Degrees of freedom | p-value | Lack of fit | F-value | R^2^ | Q^2^ | Validity | Reproducibility |
| --- | --- | --- | --- | --- | --- | --- | --- | --- |
| HA loss | 13 | 1.4e-6 | 0.15 | 20.0 | 0.81 | 0.67 | 0.53 | 0.96 |
| HA yield | 17 | 5.6e-5 | 0.52 | 12.1 | 0.82 | 0.55 | 0.84 | 0.85 |

1. Vicente T, Peixoto C, Carrondo MJT and Alves PM, Purification of recombinant baculoviruses for gene therapy using membrane processes. *Gene Ther* **16**: 766-775 (2009).

2. L E, Design of experiments : principles and applications, in Umetrics Academy - training in multivariate technology, in Umetrics AB, Umea Stockholm (2000).
